# Deoxycorticosterone Reshapes CD8^+^ T Cell State and Effector Function Through Distinct Metabolic Signaling Programs

**DOI:** 10.3390/biomedicines14102347

**Published:** 2026-10-15

**Authors:** Tao Li, Anqi Chen, Siqi Zhou, Gang Xue

**Affiliations:** 1 National Key Laboratory of Immunity and Inflammation, Suzhou Institute of Systems Medicine, Chinese Academy of Medical Sciences & Peking Union Medical College, Suzhou 215123, China; 2 Hospital for Skin Diseases, Institute of Dermatology, Chinese Academy of Medical Sciences & Peking Union Medical College, Nanjing 210042, China

**Keywords:** deoxycorticosterone, CD8^+^ T cells, T cell exhaustion, immunometabolism, metabolic signaling

## Abstract

**Background/Objectives**: Whether individual endogenous corticosteroids exert distinct immunoregulatory effects on CD8^+^ T cells remains poorly understood. We investigated how deoxycorticosterone (DOC) affects CD8^+^ T cell state and function. **Methods**: Primary mouse CD8^+^ T cells were activated with anti-CD3/CD28 and IL-2 in the presence or absence of DOC. Flow cytometry was used to assess exhaustion-associated markers and effector function, and pharmacological perturbations were used to examine the involvement of AMP-activated protein kinase (AMPK), mechanistic target of rapamycin (mTOR), and protein kinase B (AKT) signaling. **Results**: DOC promoted an exhaustion-associated phenotype characterized by increased frequencies of PD-1^+^LAG-3^+^ and PD-1^+^TIM-3^+^ cells and elevated TOX expression. DOC concurrently impaired effector function, reducing IFN-γ/TNF co-production, granzyme B expression, and IL-2 production. Pharmacological inhibition of AMPK or activation of mTOR partially attenuated exhaustion-associated features induced by DOC, whereas AKT activation partially restored effector function. **Conclusions**: DOC directly reshapes CD8^+^ T cell state and function in vitro, with exhaustion-associated and effector responses exhibiting distinct pharmacological signaling dependencies. These findings identify DOC as a previously underappreciated endogenous corticosteroid capable of modulating CD8^+^ T cell biology.

## 1. Introduction

CD8^+^ T cells are major effectors of antitumor immunity [1,2,3]. However, their functional capacity is not determined solely by antigen recognition but is continuously shaped by signals encountered within the tissue microenvironment [4]. These signals include metabolic and hormonal cues that influence T cell activation, adaptation, and effector activity [5]. Persistent exposure to adverse environmental signals can drive CD8^+^ T cells toward dysfunctional states characterized by sustained expression of inhibitory receptors and exhaustion-associated transcriptional regulators, together with progressive loss of cytokine production and cytotoxic capacity [6,7]. Defining the environmental signals that instruct these state transitions is therefore central to understanding how T cell immunity is regulated and ultimately compromised [8].

Steroid hormone signaling is an important component of this regulatory network [9]. Corticosteroids can influence T cell activation, differentiation, metabolism, and survival [10]. Yet corticosteroids comprise a structurally and functionally diverse family of endogenous molecules, raising the possibility that T cells may respond not simply to corticosteroid exposure per se, but to the identity of the individual steroid signal. Although corticosteroids are widely recognized for their immunoregulatory effects, their actions on T cells are highly context dependent [11]. Recent studies have shown that endogenous glucocorticoid signaling increases during the transition of CD8^+^ tumor-infiltrating lymphocytes toward a dysfunctional state and promotes the expression of checkpoint receptors and dysfunction-associated genes, while genetic disruption of glucocorticoid receptor signaling in CD8^+^ T cells improves effector differentiation and limits tumor progression, supporting endogenous glucocorticoid signaling as an important regulator of CD8^+^ T cell dysfunction [12]. These findings establish that endogenous steroid signals can participate directly in shaping T cell state but leave unresolved a broader question: whether individual endogenous corticosteroids convey distinct immunoregulatory information to CD8^+^ T cells.

Deoxycorticosterone (DOC; 11-deoxycorticosterone) is an endogenous corticosteroid produced as part of adrenal steroidogenesis [13,14]. DOC is present in the circulation under physiological conditions, and its circulating levels are regulated by endocrine signals, including adrenocorticotropic hormone and changes in steroid biosynthesis [15]. DOC is classically recognized for its mineralocorticoid activity, but its immunological functions remain largely unexplored compared with those of glucocorticoids and aldosterone. This gap is particularly relevant because corticosteroid signaling can intersect with metabolic and inflammatory programs in immune cells, and these effects vary according to both hormone identity and cellular context [16]. Although aldosterone signaling has been linked to T cell activation and CD8^+^ T cell cytokine production [17], whether DOC itself is sensed as an immunoregulatory cue by CD8^+^ T cells and whether it differentially affects T cell state and functional capacity remains unknown. Resolving this question could therefore extend the prevailing view of corticosteroids from a broadly immunoregulatory hormone class toward a more specific framework in which individual endogenous steroids encode distinct effects on T cell biology.

Here, we identify DOC as an endogenous corticosteroid signal that directly reshapes CD8^+^ T cell state and effector function. DOC exposure during activation promoted an exhaustion-associated state while concomitantly compromising cytokine production and cytotoxic effector programs. Importantly, these two dimensions of the DOC response exhibited distinct signaling dependencies: modulation of AMP-activated protein kinase (AMPK)–mechanistic target of rapamycin (mTOR) signaling preferentially attenuated exhaustion-associated features, whereas protein kinase B (AKT) activation partially restored effector competence. Together, our findings reveal a previously underappreciated immunoregulatory activity of DOC and support a broader framework in which corticosteroid identity, rather than corticosteroid exposure alone, contributes to the specification of CD8^+^ T cell state and function.

## 2. Materials and Methods

### 2.1. Mice

C57BL/6J mice of both sexes (6–8 weeks old) were obtained from GemPharmatech (Nanjing, China). Animals were maintained under specific pathogen-free conditions with a 12 h light/dark cycle, controlled temperature (22–24 °C) and humidity (50–60%), with ad libitum access to food and water. All animal procedures were approved by the Institutional Animal Care and Use Committee (IACUC) of the Suzhou Institute of Systems Medicine, Chinese Academy of Medical Sciences and Peking Union Medical College (protocol code ISM-IACUC-20240045).

### 2.2. Isolation and Activation of Primary Mouse CD8^+^ T Cells

Murine naïve CD8^+^ T cells were isolated from the spleens and lymph nodes of 6–8-week-old C57BL/6 mice using a naïve CD8^+^ T cell isolation kit (STEMCELL Technologies, Vancouver, BC, Canada; Cat#19858) and activated with plate-bound anti-mouse CD3 (2 μg/mL; BioLegend, San Diego, CA, USA; Cat#100340) and anti-mouse CD28 antibodies (2 μg/mL; BioLegend, Cat#102116). Purified CD8^+^ T cells were cultured in RPMI-1640 medium (Gibco, Waltham, MA, USA) supplemented with 10% fetal bovine serum (Sigma, St. Louis, MO, USA), 1% GlutaMAX (Gibco), 50 μM β-mercaptoethanol (Gibco), 1% penicillin–streptomycin (Gibco), and 100 U/mL recombinant human IL-2 (PeproTech, Cranbury, NJ, USA) at 37 °C in a humidified incubator containing 5% CO_2_. DOC (MedChemExpress, Monmouth Junction, NJ, USA; Cat#HY-113414) was added at a final concentration of 5 nM at the initiation of T cell activation (day 0) and again on day 3. The 5 nM concentration was selected as a nanomolar exposure within the range of circulating DOC concentrations reported under conditions associated with elevated DOC levels [18,19]. Control cells received an equivalent volume of dimethyl sulfoxide (DMSO) at the corresponding time points and were otherwise cultured under identical conditions.

### 2.3. Pharmacological Modulation of AMPK, mTOR, and AKT Signaling

To perturb metabolic and signaling pathways implicated in the response to DOC, DOC-treated CD8^+^ T cells were treated on day 4 of activation with the AMPK inhibitor dorsomorphin (DOR; 20 nM; MedChemExpress, Cat#HY-13418A) [20], the mTOR activator MHY1485 (100 nM; MedChemExpress, Cat#HY-B0795) [21], or the AKT activator SC79 (1 µM; MedChemExpress, Cat#HY-18749) [22]. Cells were maintained in the presence of the respective compounds for 24 h before downstream analyses. For assessment of exhaustion-associated markers, cells treated with dorsomorphin or MHY1485 were harvested on day 5 and analyzed by flow cytometry. For evaluation of effector function, SC79-treated cells were harvested after 24 h of treatment and restimulated with a stimulation cocktail (BioLegend, Cat#423304) before intracellular staining for cytokines and effector molecules. The stimulation cocktail was prepared according to the manufacturer’s instructions. Vehicle-treated cells received an equivalent volume of DMSO and were processed in parallel.

### 2.4. Flow Cytometry

Single-cell suspensions were prepared from murine CD8^+^ T cells cultured in vitro for 5 days. Cells were stained in PBS at 4 °C in the dark for 15 min with fluorochrome-conjugated surface antibodies and a fixable LIVE/DEAD viability dye. After washing with PBS, cells were resuspended in PBS containing 2% FBS for acquisition. For transcription factor staining, cells were first labeled with surface antibodies, fixed and permeabilized using the Foxp3/Transcription Factor Staining Buffer Set (Invitrogen, Carlsbad, CA, USA; Cat#00-5523-00), and subsequently stained with fluorochrome-conjugated antibodies against transcription factors following the manufacturer’s protocol. For intracellular cytokine and effector molecule analysis, cells were first restimulated with the indicated stimulation cocktail (BioLegend, Cat#423304) and subsequently fixed and permeabilized using BD Cytofix/Cytoperm Fixation/Permeabilization Kit (BD Biosciences, Franklin Lakes, NJ, USA, Cat#554714). Intracellular staining was then performed according to the manufacturer’s instructions. The following fluorochrome-conjugated antibodies were used: anti-mouse CD8α-RY703 (clone 5H10-1; BD, Cat#770124), anti-mouse PD-1-APC (clone J43; eBioscience, San Diego, CA, USA; Cat#17-9985-82), anti-mouse TIM-3-FITC (clone RMT3-23; eBioscience, Cat#11-5870-82), anti-mouse LAG-3-eFluor 450 (clone eBioC9B7W; eBioscience, Cat#48-2231-82), anti-mouse TOX-PE (clone TXRX10; eBioscience, Cat#12-6502-82), anti-mouse IFN-γ-APC (clone XMG1.2; eBioscience, Cat#17-7311-82), anti-mouse TNF-PE (clone MP6-XT22; BD Pharmingen, San Diego, CA, USA; Cat#554419), anti-mouse granzyme B (GZMB)-FITC (clone NGZB; eBioscience, Cat#11-8898-82), and anti-mouse IL-2-eFluor 450 (clone JES6-5H4; eBioscience, Cat#48-7021-82). Only viable singlet CD8^+^ T cells were included in downstream analyses. Flow cytometry data were acquired using a BD LSRFortessa (BD Biosciences, Franklin Lakes, NJ, USA) and analyzed with FlowJo v10.8.1 (BD Life Sciences, Ashland, OR, USA).

### 2.5. Statistical Analysis

Data are presented as mean ± SD. Each experimental condition was analyzed in three parallel cultures. Statistical analyses were performed using GraphPad Prism (version 10.4.2; GraphPad Software, Boston, MA, USA). Comparisons between two groups were performed using two-tailed unpaired Student’s *t*-tests when appropriate. For experiments involving more than two groups, one-way ANOVA followed by Tukey’s multiple-comparison test was used. All statistical tests were two-sided, and *p* < 0.05 was considered statistically significant.

## 3. Results

### 3.1. Deoxycorticosterone Reshapes CD8^+^ T Cell State and Compromises Effector Function

**Figure 1:**
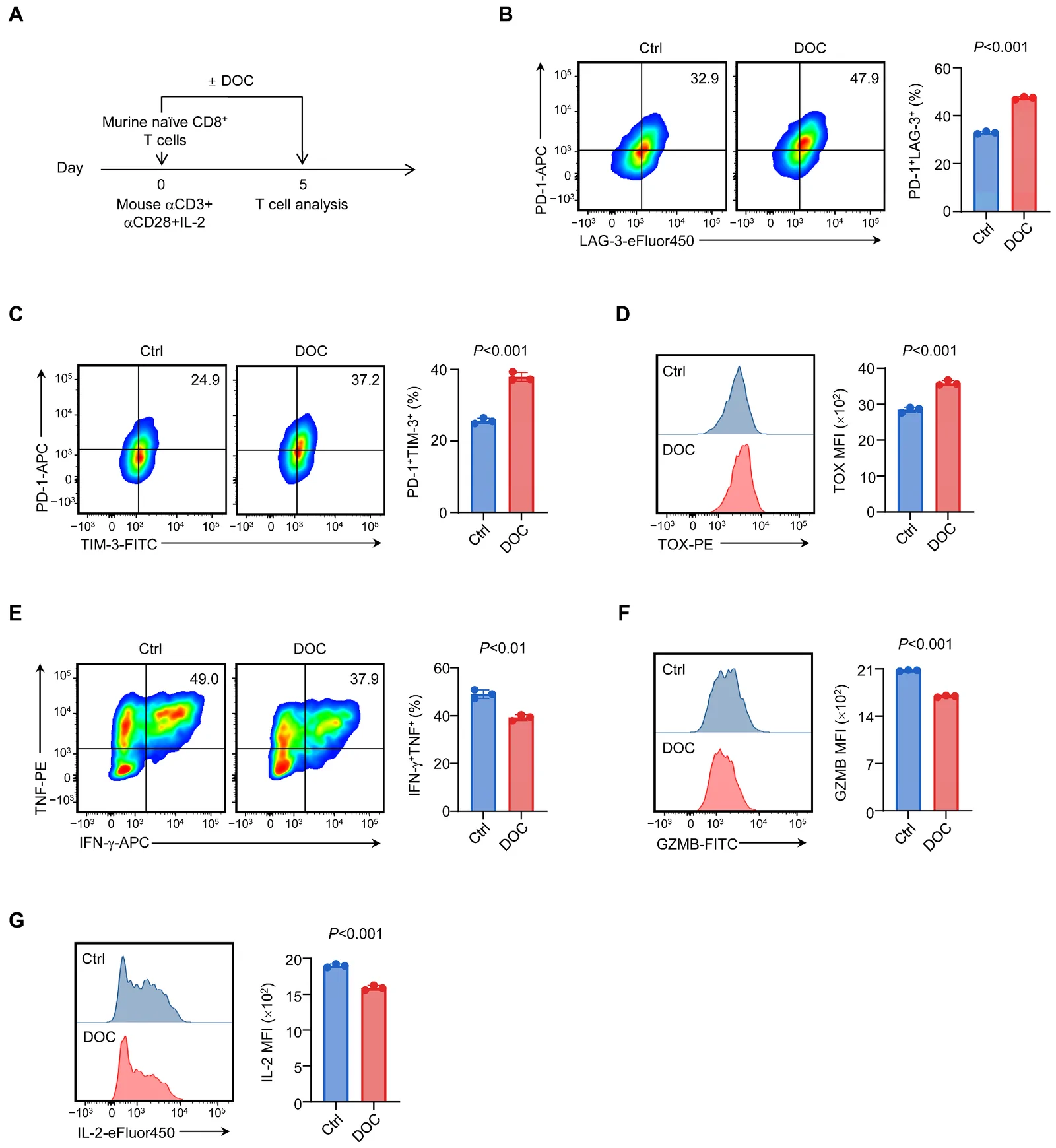
Deoxycorticosterone reshapes CD8^+^ T cell state and compromises effector function. Primary mouse CD8^+^ T cells were activated with anti-CD3 and anti-CD28 in the presence of IL-2 and treated with vehicle control (Ctrl) or 5 nM deoxycorticosterone (DOC) for 5 days. (**A**) Schematic of the experimental design. (**B**) Representative flow cytometry plots and quantification of the frequency of PD-1^+^LAG-3^+^ CD8^+^ T cells. (**C**) Representative flow cytometry plots and quantification of the frequency of PD-1^+^TIM-3^+^ CD8^+^ T cells. (**D**) Representative flow cytometry histogram and quantification of TOX expression. (**E**) Representative flow cytometry plots and quantification of the frequency of IFN-γ^+^TNF^+^ CD8^+^ T cells following restimulation with stimulation cocktail. (**F**) Representative flow cytometry histograms and quantification of granzyme B (GZMB) expression following restimulation. (**G**) Representative flow cytometry histograms and quantification of IL-2 expression following restimulation. Data are presented as mean ± SD (*n* = 3). Data shown are from one representative experiment of two independent experiments with similar results. Statistical significance was determined using a two-tailed unpaired Student’s *t*-test. Statistical significance is indicated in the figure.

To determine how deoxycorticosterone (DOC) affects CD8^+^ T cell state and function, we isolated naive mouse CD8^+^ T cells and activated them with anti-CD3 and anti-CD28 in the presence of IL-2, with or without 5 nM DOC (Figure 1A). We first examined whether DOC alters the acquisition of exhaustion-associated features, a defining dimension of dysfunctional CD8^+^ T cell states [23]. Flow cytometric analysis revealed that DOC treatment increased the frequency of PD-1^+^LAG-3^+^ CD8^+^ T cells (Figure 1B) and PD-1^+^TIM-3^+^ CD8^+^ T cells (Figure 1C). We next assessed the expression of TOX, a transcriptional regulator associated with T cell exhaustion [24]. Consistent with the acquisition of an exhaustion-associated phenotype, DOC treatment also increased TOX expression (Figure 1D). Thus, exposure to DOC during activation was sufficient to shift CD8^+^ T cells toward an exhaustion-associated state.

We next determined whether this phenotypic remodeling was accompanied by a loss of functional competence. Dysfunctional CD8^+^ T cells progressively lose cytokine production and cytotoxic capacity [25]. Following restimulation, DOC-treated cells exhibited a lower frequency of polyfunctional IFN-γ^+^TNF^+^ cells than control cells (Figure 1E). This functional impairment extended beyond cytokine co-production, as DOC also reduced granzyme B (GZMB) expression (Figure 1F) and IL-2 production (Figure 1G). Together, these results demonstrate that DOC coordinately remodels CD8^+^ T cell state and effector function, coupling the acquisition of exhaustion-associated features with a broad reduction in effector competence.

### 3.2. AMPK–mTOR Signaling Contributes to the Exhaustion-Associated Response to Deoxycorticosterone

We next investigated the signaling pathways underlying the DOC-induced exhaustion-associated phenotype. Metabolic signaling is closely linked to CD8^+^ T cell differentiation and functional state, and metabolic dysfunction can emerge early during T cell exhaustion [26]. In particular, AMPK and mTOR are key regulators of T cell metabolic programs [27,28], making the AMPK–mTOR axis a plausible signaling node through which corticosteroid-associated metabolic cues may influence T cell state [29,30].

**Figure 2:**
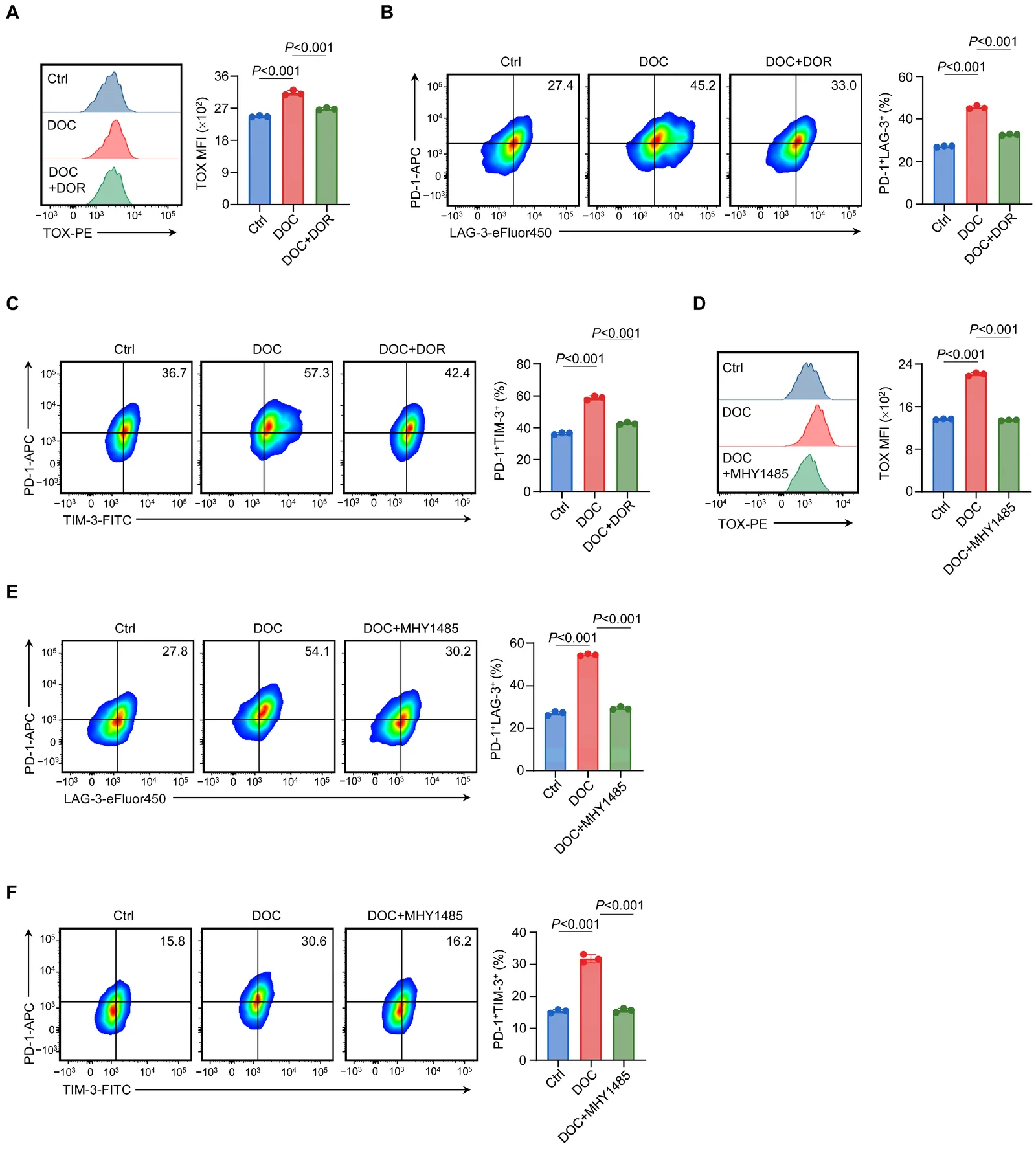
AMP-activated protein kinase (AMPK)–mechanistic target of rapamycin (mTOR) signaling contributes to the exhaustion-associated response to deoxycorticosterone. Primary mouse CD8^+^ T cells were activated and cultured in the presence of vehicle control (Ctrl) or 5 nM deoxycorticosterone (DOC), with or without the AMPK inhibitor dorsomorphin (DOR, 20 nM) or the mTOR activator MHY1485 (100 nM), as indicated. (**A**–**C**) Representative flow cytometry histograms or plots and quantification of TOX expression (**A**), the frequency of PD-1^+^LAG-3^+^ cells (**B**), and the frequency of PD-1^+^TIM-3^+^ cells (**C**) in Ctrl, DOC, and DOC+DOR groups. (**D**–**F**) Representative flow cytometry histograms or plots and quantification of TOX expression (**D**), the frequency of PD-1^+^LAG-3^+^ cells (**E**), and the frequency of PD-1^+^TIM-3^+^ cells (**F**) in Ctrl, DOC, and DOC+MHY1485 groups. Data are presented as mean ± SD (*n* = 3). Data shown are from one representative experiment of two independent experiments with similar results. Statistical significance was determined by one-way ANOVA followed by Tukey’s multiple-comparison test. Statistical significance is indicated in the figure.

To determine whether AMPK–mTOR signaling contributes to the DOC-induced exhaustion-associated phenotype, we pharmacologically inhibited AMPK with dorsomorphin (DOR) or activated mTOR with MHY1485. DOR partially attenuated the DOC-induced increase in TOX expression (Figure 2A). Similarly, DOR reduced the frequency of PD-1^+^LAG-3^+^ cells in DOC-treated cultures (Figure 2B). DOR treatment also partially reduced the frequency of PD-1^+^TIM-3^+^ cells (Figure 2C). Pharmacological activation of mTOR with MHY1485 also partially attenuated the DOC-induced increase in TOX expression (Figure 2D), the frequency of PD-1^+^LAG-3^+^ cells (Figure 2E), and the frequency of PD-1^+^TIM-3^+^ cells (Figure 2F). Together, these findings indicate that AMPK–mTOR signaling contributes to the DOC-induced exhaustion-associated phenotype in CD8^+^ T cells.

### 3.3. AKT Activation Partially Restores Effector Function Impaired by Deoxycorticosterone

**Figure 3:**
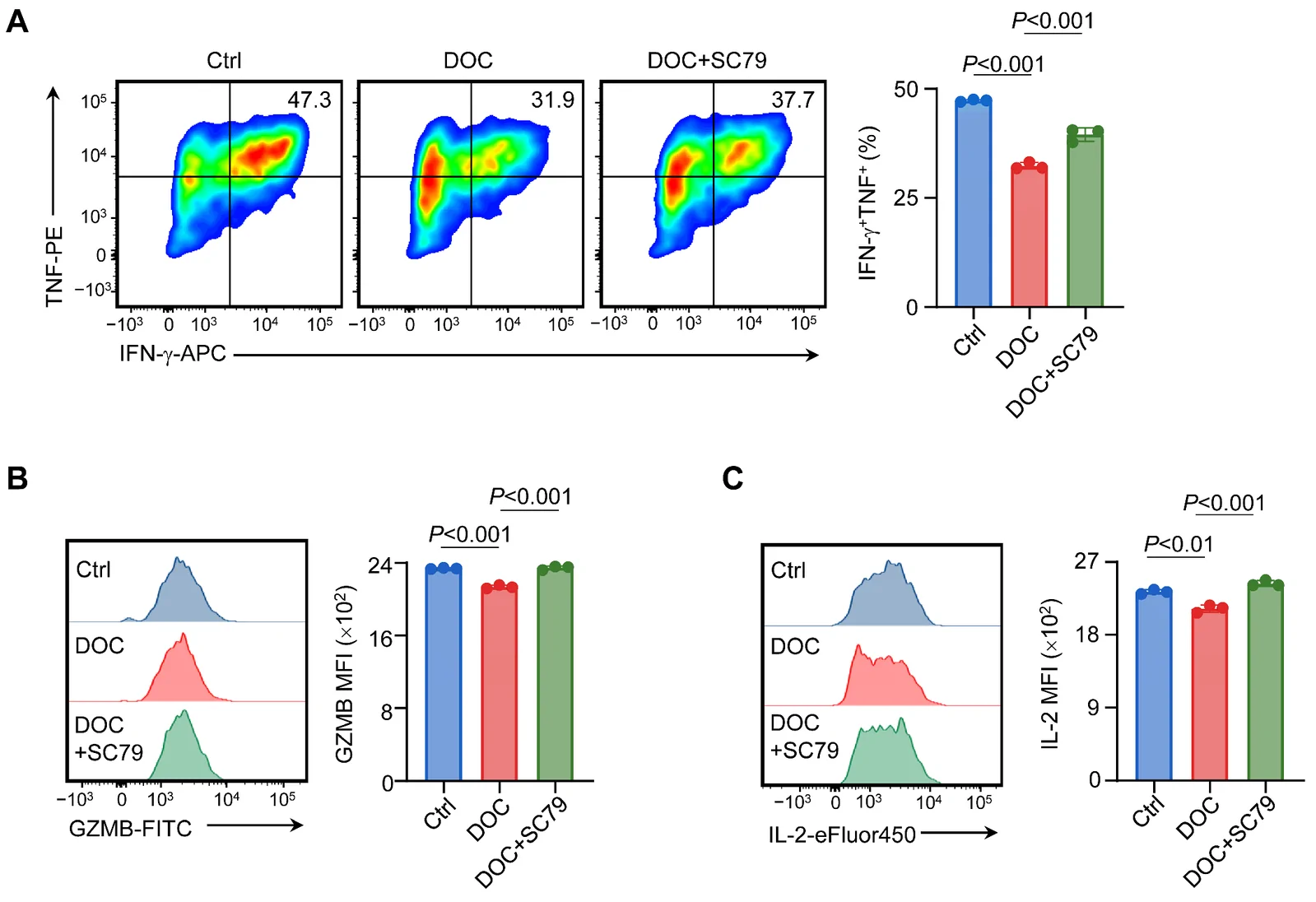
Protein kinase B (AKT) activation partially restores effector function impaired by deoxycorticosterone. Primary mouse CD8^+^ T cells were activated and cultured in the presence of vehicle control (Ctrl) or 5 nM DOC. DOC-treated cells were additionally exposed to the AKT activator SC79 (1 µM), followed by restimulation and flow cytometric analysis of effector function. (**A**) Representative flow cytometry plots and quantification of the frequency of IFN-γ^+^TNF^+^ cells. (**B**,**C**) Representative flow cytometry histograms and quantification of GZMB (**B**) and IL-2 (**C**) expression. Data are presented as mean ± SD (*n* = 3). Data shown are from one representative experiment of two independent experiments with similar results. Statistical significance was determined by one-way ANOVA followed by Tukey’s multiple-comparison test. Statistical significance is indicated in the figure.

Because AKT signaling is a central regulator of CD8^+^ T cell effector responses and supports cytokine production and cytotoxic effector programs [31,32], we next examined whether pharmacological activation of AKT could counteract the functional impairment induced by DOC. Treatment with the AKT activator SC79 partially restored the frequency of IFN-γ^+^TNF^+^ cells reduced by DOC exposure (Figure 3A). SC79 also increased intracellular GZMB (Figure 3B) and IL-2 expression (Figure 3C) in DOC-treated cells. Together, these findings indicate that AKT signaling contributes to the loss of effector competence induced by DOC.

## 4. Discussion

Our findings identify deoxycorticosterone as an endogenous corticosteroid signal that can directly reshape CD8^+^ T cell state and function. DOC exposure during T cell activation shifted CD8^+^ T cells toward an exhaustion-associated state while concomitantly compromising their effector competence. These effects were observed in purified primary CD8^+^ T cells, indicating that DOC can act directly on T cells without requiring other immune or tumor-derived cells. Our findings extend previous work showing that endogenous glucocorticoid signaling contributes to CD8^+^ T cell dysfunction [12]. Importantly, they broaden this paradigm beyond glucocorticoids and raise the possibility that endogenous corticosteroids should not be viewed as a functionally uniform class of immunoregulatory signals. Instead, the identity of the corticosteroid itself may represent an additional layer of information through which endocrine environments shape T cell biology.

This concept is particularly relevant because the corticosteroid milieu encountered by immune cells is neither static nor compositionally uniform. Glucocorticoid signaling has been shown to promote checkpoint receptor expression and dysfunctional transcriptional programs in CD8^+^ T cells, and disruption of glucocorticoid receptor signaling can improve effector differentiation and tumor control [12]. Corticosteroid signals can also interact with cellular metabolic programs that shape T cell function [33]. Our results extend this framework to DOC, an endogenous corticosteroid whose immunological activity has received comparatively little attention. Rather than merely altering a single activation parameter, DOC affected two fundamental dimensions of CD8^+^ T cell biology: phenotypic state and effector capacity. This observation suggests that the composition of the endogenous corticosteroid environment—not simply the presence or absence of corticosteroid signaling—may influence how CD8^+^ T cells adapt to their surroundings. Whether other endogenous corticosteroids impose overlapping or distinct T cell programs will be important to determine.

A second implication of our findings is that the phenotypic and functional dimensions of the DOC response exhibit differential signaling dependencies. Metabolic remodeling is closely linked to CD8^+^ T cell differentiation and dysfunction, and metabolic defects can emerge early during exhaustion [34]. AMPK and mTOR are central regulators of cellular energy balance and anabolic metabolism and are important determinants of T cell differentiation and function [29]. In our experiments, inhibition of AMPK or activation of mTOR partially reduced DOC-induced TOX and inhibitory receptor expression, linking the AMPK–mTOR axis to the exhaustion-associated state elicited by DOC. Although neither intervention fully restored the control phenotype, the consistent effects of complementary AMPK and mTOR modulation support the functional involvement of this signaling axis in the DOC response. Because these experiments relied on pharmacological perturbation, the precise directionality and molecular organization of this relationship remain to be established.

The signaling requirements associated with loss of effector competence were notably different. Activation of AKT partially restored cytokine production, granzyme B expression, and IL-2 production in DOC-treated CD8^+^ T cells. The PI3K–AKT pathway is a central regulator of T cell activation and effector function and integrates TCR and co-stimulatory signals with metabolic programs [35,36]. The coordinated improvement of multiple effector readouts following AKT activation therefore identifies AKT-dependent signaling as a functionally relevant component of the DOC response. Importantly, when considered together with the AMPK–mTOR experiments, these findings reveal a division in the signaling architecture associated with DOC-induced remodeling: AMPK–mTOR modulation preferentially affected exhaustion-associated phenotypic features, whereas AKT activation preferentially restored effector competence. Although these pharmacological experiments do not establish that the pathways operate independently, they argue against viewing DOC-induced dysfunction as a single, indivisible cellular program. Instead, phenotypic state and functional capacity appear to represent related but differentially regulatable dimensions of the response to an endogenous corticosteroid signal.

This distinction may have broader implications for how environmentally induced T cell dysfunction is conceptualized [37]. Acquisition of exhaustion-associated features and loss of effector activity frequently occur together, yet their coexistence does not necessarily imply that they arise through an identical signaling program. Our observations with DOC provide an example in which these dimensions can be differentially modified pharmacologically. Thus, endocrine cues may reshape T cell behavior through a modular signaling architecture in which cell-state remodeling and functional competence are coordinated but not obligatorily coupled. Defining how these modules intersect and whether similar organization applies to other endogenous hormonal signals could provide a more nuanced framework for understanding the environmental control of T cell states.

Several limitations define the scope of these conclusions. The present study establishes the direct effects of DOC in an in vitro primary CD8^+^ T cell system and uses pharmacological perturbations to identify signaling dependencies associated with the resulting phenotype. Accordingly, the molecular mechanisms by which DOC is sensed and engages these signaling networks, including the receptor-level and transcriptional mechanisms underlying its effects, as well as their relevance in more physiologically complex settings, remain to be fully defined. Comparative transcriptomic profiling of DOC- and classical glucocorticoid-treated CD8^+^ T cells, together with assessment of GR occupancy at relevant regulatory regions, may help distinguish shared from steroid-specific transcriptional responses. Whether the effects of DOC observed in purified CD8^+^ T cells are recapitulated following systemic DOC exposure in vivo remains to be established. Moreover, the exhaustion-associated phenotype observed here should not be equated with the fully established exhaustion states that emerge during chronic antigen exposure in tumors or persistent infection. These considerations define the scope of the present findings while providing a basis for further investigation of DOC-mediated regulation of T cell biology.

In summary, our study identifies DOC as a previously underappreciated endogenous regulator of CD8^+^ T cell state and effector function and reveals differential signaling dependencies underlying these two dimensions of the response. More broadly, these findings support a model in which corticosteroid identity itself may contribute to the information encoded by the endocrine microenvironment, while metabolic signaling determines how that information is translated into T cell state and function. This framework expands the view of corticosteroids from broadly immunoregulatory hormones toward distinct endogenous cues capable of differentially shaping T cell biology.

## Data Availability

Data presented in this study are available in the article. Further inquiries can be directed to the corresponding author.

## Abbreviations

The following abbreviations are used in this manuscript:

DOC	Deoxycorticosterone
DOR	Dorsomorphin
AMPK	AMP-activated protein kinase
mTOR	mechanistic target of rapamycin
AKT	protein kinase B
